# Antioxidant and Inflammation-Attenuating Ability of Human Milk, Infant Formulas and Their Oligosaccharides †

**DOI:** 10.3390/foods14060960

**Published:** 2025-03-11

**Authors:** Andrea Leong, Christopher Pillidge, Harsharn Gill

**Affiliations:** School of Science, RMIT University, Melbourne, VIC 3083, Australia; andrea.leong@rmit.edu.au (A.L.); harsharn.gill@rmit.edu.au (H.G.)

**Keywords:** infant formula, milk oligosaccharides, antioxidant, cytokine gene expression, inflammation, cell culture model, biochemical indices

## Abstract

Human milk (HM) provides maximum health benefits for infants. This is due in part to the activity of its minor components, including HM proteins and oligosaccharides (HMOs). Where HM is unavailable, infant formulas (IFs) are normally used, the two most common types being cow milk- and goat milk-based formulas (CIF and GIF). The aim of this study was to compare the antioxidant properties of HM, CIF and GIF, together with those of their respective oligosaccharides (HMOs, CMOs and GMOs), using in vitro model systems. The ability of these oligosaccharides to attenuate inflammation (expression of IL-1α, TNFα, IL-6 and IL-8) was also assessed using a U937 cell culture model. Results showed that GIF and GMOs exhibited the highest antioxidant potential. The iron-reducing and iron-chelating properties of both IFs were comparable to those for HM, while the iron-chelating ability of the CMOs was lowest. None of the oligosaccharides significantly reduced U937 cytokine expression following induction of inflammation; however, GMOs consistently reduced expression of IL-1α, TNFα and IL-6 to a greater extent than the other oligosaccharides, presumably by competitive binding to immune receptors. In conclusion, GMOs have a greater antioxidant potential than CMOs and may have some inflammation-attenuating ability as well.

## 1. Introduction

Human milk (HM) contains a multitude of components essential for infant growth and development [1]. HM oligosaccharides (HMOs), the third most abundant solid component after fat and lactose, are critical elements in controlling the development of the infant gut microbiota and helping protect against pathogens [2,3,4]. Further, HMOs are strongly bifidogenic, supporting the growth of these and other bacteria including probiotics via their selective uptake as growth substrates, and this effect is known to be strain-specific [5,6].

There are over 200 distinct types of HMOs in HM with varying molecular structures [7,8]. Numerous studies (both human and in vitro) are unraveling the complex interactions between HMOs and the infant gut epithelial and immune cells (immunomodulatory properties) including controlling development of the infant gut microbiota and reducing the impact of pathogens [3,8,9,10,11]. In addition, HM and HMOs have significant antioxidant functions through a variety of mechanisms, providing further health benefits to the infant [12,13,14].

When breastfeeding is not possible, infant formulas (IFs) are commonly used [1]. However, little is known about the comparative benefits of HMOs in breastmilk compared with oligosaccharides present in IFs [8], even though a small number of food-regulated synthetic HMOs may be added in IF formulations—notably 2′-fucosyllactose (2′-FL) and lacto-N-neotetraose (LNnT), which are permitted in Australia and most other countries [15]. Moreover, although cow milk-based IFs are by far the most popular due to their scale of production [16], other mammalian IFs such as goat’s milk-based IFs (GIF) are also available in the market. The abundance and diversity of oligosaccharides in other mammalian milks (and IFs derived from them) is notably far less than that observed in HM [16]. Nevertheless, two earlier studies have shown that goat’s milk oligosaccharides can reduce inflammation in rat models with induced colitis [17,18]. The ability of galacto-oligosaccharides (GOS) to alleviate damage to the intestinal barrier and reduce inflammatory responses in lipopolysaccharide (LPS)-challenged mice (the mice were intra-peritoneally challenged with LPS after intragastric administration of GOS) has also been reported [19].

An understanding of the comparative benefits of oligosaccharides in IFs from different animal sources versus those in HM is essential for developing future IFs with optimal formulations. In this study, we investigated the in vitro antioxidant and inflammation-attenuating properties of oligosaccharides extracted from HM and two IFs (one goat- and one cow-milk based). Our aim was to see which type of oligosaccharide provided the maximum health benefits based on in vitro biochemical antioxidant and immune assays. Such information may help lead to the selection of new or better oligosaccharides in IF formulations.

## 2. Materials and Methods

### 2.1. Human Milk and Infant Formula Samples and Oligosaccharide Extraction

Human milk (HM) samples were donated by breastfeeding mothers following approval by the RMIT University Human Research Ethics Committee (Approval number 55-19/22280). Four HM samples were obtained, pooled and aliquots frozen at −80 °C until analyzed. Cow’s milk infant formula (CIF) (S26^®^ Original Stage 1, Wyeth Nutrition, Baulkham Hills, NSW, Australia) and goat’s milk infant formula (GIF) (Oli6^®^ Stage 1 Goat’s Milk Infant, Nuchev Pty. Ltd., Melbourne, VIC, Australia) were obtained locally and reconstituted according to manufacturer’s instructions. Neither IF had specific oligosaccharides added to them as part of the formulation.

Native oligosaccharide fractions from HM (HMO), goat’s milk IF (GMO) and cow’s milk IF (CMO) were extracted using the following protocol [20,21]: milk samples were centrifuged at 4000× *g* for 30 min at 4 °C to remove milk lipids. Defatted milk was then centrifuged through a 10 kDa MWCO filter (Amicon^®^ Centrifugal Filters, Merck, Macquarie Park, NSW 2113, Australia) (4000× *g* 30 min) with a regenerated cellulose membrane, and the filtrate kept frozen at −80 °C until assayed. Sterile water was used as the negative control in all assays.

### 2.2. Antioxidant Assays

#### 2.2.1. 2,2-Diphenyl-1-picrylhydrazyl (DPPH) Radical Scavenging Assay

The DPPH radical scavenging assay, which is widely used to indicate antioxidant properties [22], was conducted as described in previous studies [23,24] with minor modifications. Briefly, 100 μL of the sample was added to 1 mL of methanolic DPPH solution (0.1 mM), and the solution was incubated in a water bath at 37 °C for 30 min in the dark. Absorbance was measured at 517 nm. A linear calibration curve using Trolox^®^ at concentrations (0.01, 0.02, 0.04, 0.08 and 0.1 mg/L) was prepared for quantitation). Methanolic DPPH solution was used as the control. DPPH radical scavenging ability was calculated based on the following formula:

$$\%\;\mathrm{DPPH}\;\mathrm{scavenging}\;\mathrm{ability}=\frac{\left({\mathrm{Abs}}_{\mathrm{control}}-{\mathrm{Abs}}_{\mathrm{sample}}\right)}{{\mathrm{Abs}}_{\mathrm{control}}}\times 100\%$$

#### 2.2.2. Hydroxyl Radical Scavenging Ability

Measurement of hydroxyl radical scavenging ability was conducted as described by Zhu et al. [25]. Briefly, 1 mL of O-phenanthroline (2.5 mM), 1 mL of phosphate-buffered saline (PBS) (0.4 M, pH 7.4), 1 mL FeSO_4_ (2.5 mM) and 0.5 mL H_2_O_2_ (0.01%) were added to 1.5 mL milk or oligosaccharide samples. The reaction between H_2_O_2_ and Fe^2+^ produces highly reactive hydroxyl radicals (Fenton Reaction) [26]. The mixture was incubated at room temperature for 5 min and absorbance was measured at 536 nm. Hydroxyl radical scavenging ability was measured based on the following formula:

$$\mathrm{Scavenging}\;\mathrm{ability}\;\left(\%\right)=\frac{A_{2}-A_{1}}{A_{0}-A_{1}}\times 100\%$$

where *A*_0_ refers to the absorbance of the control (without milk and H_2_O_2_); *A*_1_ refers to the absorbance of the blank (milk sample replaced with water); *A*_2_ refers to the absorbance of test samples (milk sample and H_2_O_2_).

#### 2.2.3. Superoxide Radical Scavenging Ability

The protocol was conducted as described by Ding et al. [27]. A 2.8 mL volume of Tris-HCl buffer (0.05 M, pH 8.2), plus 0.1 mL pyrogallic acid (0.05 M) and 0.1 mL sample were mixed and incubated at 25 °C for 4 min in the dark. Subsequently, 1 mL of HCl (8 M) was added to stop the reaction and absorbance measured at 320 nm. The scavenging ability was calculated based on the following formula:

$$\mathrm{Scavenging}\;\mathrm{ability}\;\left(\%\right)=\frac{A_{\mathrm{sample}}-A_{\mathrm{blank}}}{A_{\mathrm{blank}}}\times 100\%$$

#### 2.2.4. Potassium Ferricyanide Reducing Power (FER)

Iron metabolism is closely related to stress and the action of antioxidants [28]. The presence of transition metal ions such as Fe^3+^ induce lipid oxidation and formation of reactive peroxyl and alkoxyl radicals; hence, compounds that exhibit metal chelating ability can neutralize this oxidation process. This can be assessed by the ability of compounds to reduce ferric ion (Fe^3+^)-ligand complexes to ferrous (Fe^2+^) complexes. Compounds such as ferrozine and ferricyanide are commonly used in such assays as an alternative to tripyridyltriazine (TPTZ) [29].

In this study, the FER protocol was adapted from an earlier study by Liu et al. [30]. Briefly, 250 μL of sample was added to 250 μL of PBS buffer (0.2 M) and 250 μL potassium ferricyanide solution (1%). The mixture was then incubated at 50 °C in a water bath for 20 min. After cooling to room temperature, 250 µL of trichloroacetic acid (10%) was added, followed by 100 µL of ferric chloride (0.1%). The absorbance was measured at 700 nm. The strength of reducing power is indicated by higher absorbance readings.

#### 2.2.5. Fe^2+^-Chelating Ability (CA)

Fe^2+^-chelating ability was determined using the method described by Liu et al. [30]. Briefly, 100 μL of sample was mixed with 5 μL ferrous chloride (2 mM) and 20 µL ferrozine (5 mM). The mixture was vortexed to ensure proper mixing and held still for 10 min at room temperature. The absorbance of the mixture was subsequently measured at 562 nm. Fe^2+^ chelating ability was calculated based on the following formula:

$$\%\;\mathrm{chelating}\;\mathrm{abiliy}=\left[\;1-\frac{\left(A_{1}-A_{2}\right)}{A_{0}}\right]\times 100\%$$

where *A*_0_ refers to the absorbance of the control (water); *A*_1_ refers to the absorbance of the sample; *A*_2_ refers to the absorbance of the sample without added ferrozine.

### 2.3. Cell Culture Conditions and Simulation of Inflammation Conditions

U937 monocytic cells [31] from RMIT’s collection and initially sourced from the American Type Culture Collection (ATCC) (CRL-1593.2) were used as a model system to assess immune responses in the gastrointestinal tract. U937 cells (undifferentiated) were cultured in Roswell Park Memorial Institute (RPMI) 1640 media with 10% heat inactivated foetal bovine serum (FBS) and 1% of an antibiotic/antimycotic solution containing 10,000 units/mL of penicillin and 10,000 μg/mL of streptomycin (Thermo Fisher Scientific, Scoresby, VIC, Australia). Incubation conditions were 37 °C, 95% humidity with 5% carbon dioxide. At 80% confluency, cells were passaged as follows: Cells were centrifuged at 1000× *g* for 5 min. The supernatant was discarded, and the cell pellet was reconstituted with 10 mL of fresh media to a density of 10^5^ cells/mL.

Inflammatory conditions were induced in cell cultures by adding any one of the following: (1) 10 µg of *E. coli* O111:B4-derived lipopolysaccharide (LPS) (Sigma L4391); (2) *Escherichia coli* live cells; (3) *Salmonella typhimurium* live cells (both from RMIT collection, 1 × 10^7^ CFU added per assay). U937 cells were seeded at a density of 1 × 10^6^ cells and incubated with either live bacteria or LPS for 3 h and subsequently washed in sterile PBS and then treated with milk oligosaccharide fractions for a further 3 h. Water was used in place of LPS/bacteria to represent non-inflammatory conditions (controls).

### 2.4. Cytokine Expression Levels

Expression levels of pro-inflammatory cytokines (TNF-α, IL-1α, IL-6 and IL-8) and their responses to oligosaccharide fractions were determined by measuring levels of RNA transcripts [32,33]. RNA extraction from U937 cells was performed using the Aurum^TM^ total RNA mini kit (BioRad, South Granville, NSW 2142 Australia), as per the manufacturer’s instructions. RNA quantification was performed using a NanoDrop spectrophotometer at 260 nm, and RNA purity determined by measuring the absorbance ratios at 240/260 nm and 280/260 nm. cDNA conversion was performed using the iScript cDNA synthesis kit (BioRad) as per the manufacturer’s instructions. Briefly, 4 µL of Reaction mix, 1 µL reverse transcriptase, RNA template (final concentration of 1 μg) and DNAse/RNAse free water was added to form a total volume of 20 µL. The initial priming step was at 25 °C for 5 min, followed by a reverse transcription step at 46 °C for 20 min, then an inactivation step at 95 °C for 1 min. Samples were then held at 4 °C prior to quantification.

Quantification of cytokine gene transcripts (IL-1α, TNFα, IL-6 and IL-8) was performed via reverse-transcriptase quantitative polymerase chain reaction (RT-qPCR). Glyceraldehyde 3-phosphate dehydrogenase (GAPDH), a constitutively expressed housekeeping gene, was selected as the steady-state transcription control [32,33]. Primers used were as follows: GAPDH forward 5′-GGAAGGTGAAGGTCGGAGTC-3′, reverse 5′-TCAGCCTTGACGGTGCCATG-3′ [32]; TNF-α forward 5′-TCTCGAACCCCGAGTGACAA-3′, reverse 5′-TATCTCTCAGCTCCACGCCA-3′ [34]; IL-1α forward 5′-ATCATGTAAGCTATGGCCCACT-3′, reverse 5′-CTTCCCGTTGGTTGCTACTAC-3′ [35]; IL-6 forward 5′-GATGGCTGAAAAAGATGGATGC-3′, reverse 5′-TGGTTGGGTCAGGGGTGGTT-3′ [36]; IL-8 forward 5′-CATACTCCAAACCTTTCCACCC-3′, reverse 5′-CAACCCTCTGCACCCAGTTTT-3′ (this study). The correct PCR product was confirmed by gel electrophoresis, and PCR efficiencies determined from a standard curve were close to 100%. qPCR reactions were prepared with 10 µL SensiFAST™ SYBR^®^ No-ROX Mix (Bioline Pty Ltd., Gregory Hills, NSW 2557, Australia), 0.8 µL primers (10 µM forward and reverse primers, each), cDNA template and nuclease-free water made up to 20 µL. PCR conditions were as follows: 1 cycle at 95 °C for 3 min; 40 cycles at 95 °C for 30 s, 72 °C for 30 s, annealing temperature (59–63 °C depending on primers used for 30 s); then a final extension at 72 °C for 5 min followed by a holding step at 4 °C. Negative controls were prepared by substituting template DNA with nuclease-free water.

### 2.5. Experimental Design and Statistical Analysis

All analyses were performed in triplicate (*n* = 3) with results for each assay depicted as the mean ± standard error (SE). The differences between sample groups and controls were tested for significance by one-way ANOVA and, subsequently, Tukey’s post-hoc test (analyses were performed using GraphPad Prism version 7 for Windows, GraphPad Software). Statistically significant results are defined as *p* < 0.05 unless otherwise stated.

## 3. Results

### 3.1. Antioxidant Properties

The term ‘antioxidant’ denotes molecules or fractions with free radical scavenging or metal-reducing properties. These properties were determined both for the oligosaccharide fractions, as well as for whole HM and two commercial IFs (GIF and CIF).

#### 3.1.1. Free Radical Scavenging Abilities

**Figure 1:**
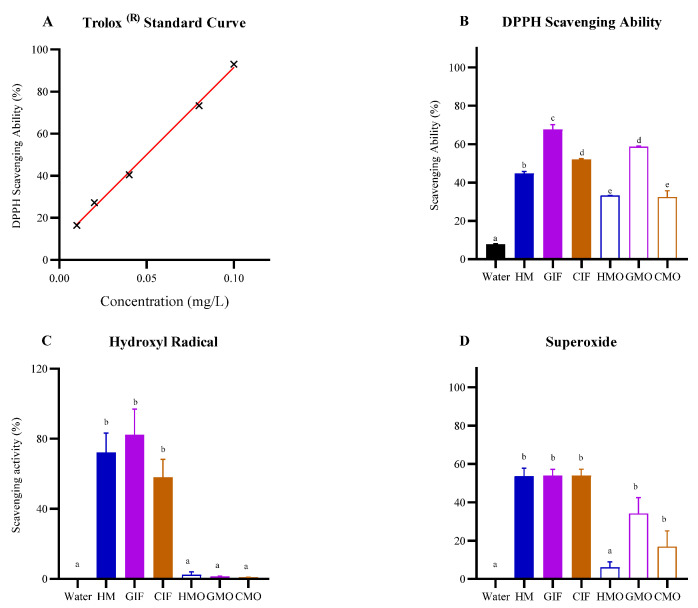
Antioxidant (reducing) properties of HM, GIF and CIF and milk oligosaccharide fractions: (**A**) Trolox standard curve used in calculating DPPH-reducing activity; (**B**) DPPH-, (**C**) hydroxyl- and (**D**) superoxide-reducing activities. The black bar in each of (**B**,**C**) or (**D**) represents the negative control (water). Bars with different superscripts (a–e) denote significant differences (*p* < 0.05) between treatments. Abbreviations: HM, human milk; GIF: reconstituted whole goat’s milk-based infant formula; CIF, reconstituted whole cow’s milk-based infant formula; HMO, human milk oligosaccharides prepared from HM; GMO, goat’s milk oligosaccharides prepared from GIF; CMO, cow’s milk oligosaccharides prepared from CIF.

These were determined using three radicals (DPPH, hydroxyl and superoxide radicals). DPPH is an organic radical which is neutralized through acceptance of electrons from antioxidants (electron donors), resulting in a change in color measured by reduction in absorbance at 517 nm. DPPH-radical scavenging was calculated relative to reduction in the water-soluble Vitamin E analog Trolox^®^ (see Section 2) and standard curve (Figure 1A; R^2^ = 0.9973). Remarkably, all three oligosaccharide fractions prepared from HM and IFs had only slightly less DPPH-scavenging ability than those seen for whole HM or IF samples (Figure 1B). GIF displayed the highest DPPH scavenging ability (68%), followed by CIF (52%) and HM (45%), and these were all statistically different from each other (HM vs. GIF, *p* < 0.0001; HM vs. CIF, *p* < 0.05; GIF vs. CIF, *p* < 0.0001). Within the oligosaccharide fractions, GMOs had the highest radical scavenging capacity (59%), while HMOs and CMOs showed capacities of 33% and 32%, respectively, versus a background level of 8% (control) (Figure 1B).

Hydroxyl radicals react with carbohydrates resulting in cleavage of glycosidic bonds. Like the results for DPPH, GIF exhibited the greatest scavenging ability (82%), followed by HM (72%) and CIF (58%) (Figure 1C). Regarding milk oligosaccharides, in contrast to the former results for DPPH, hydroxyl radical scavenging activity was equivalent to that for the controls, and in this case the background activity was negligible. This meant that any observed differences between oligosaccharide samples and controls were insignificant (*p* > 0.05) (Figure 1C).

In the superoxide radical assay, pyrogallol undergoes autoxidation in the presence of oxygen and produces superoxide radicals. Compounds that can scavenge these radicals are deemed to have antioxidant properties. All whole milk samples (HM, GIF and CIF) neutralized these radicals with efficiencies of up to 53% (results were not statistically different) (Figure 1D). Within the oligosaccharide fractions, and like the results for DPPH, the GMO fraction had maximum scavenging ability which was only slightly less than that seen for HM, GIF and CIF. HMOs showed the lowest capacity of all samples tested (*p* < 0.05) (Figure 1D).

#### 3.1.2. Iron-Reduction and Iron-Chelation Abilities

**Figure 2:**
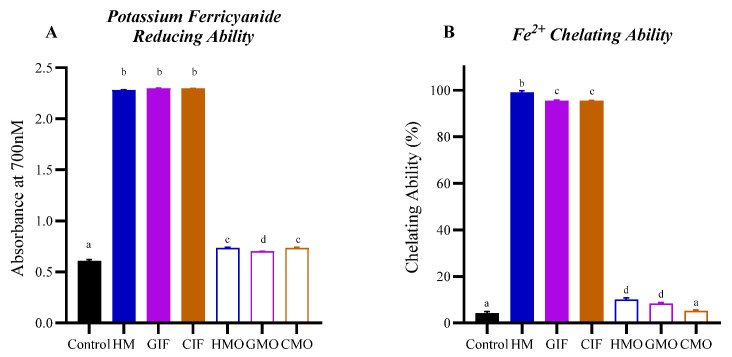
Antioxidant and Fe^2+^-chelating properties of HM, IFs and milk oligosaccharide fractions measured by (**A**) potassium ferricyanide ([Fe(CN)_6_]^3−^) reduction and (**B**) Fe^2+^-chelating ability. The black bars represent the negative control (water). Bars with different superscript letters denote significant differences between treatments (*p* < 0.05). See Figure 1 for sample abbreviations.

In this study, we used ferricyanide to measure Fe^3+^-reducing capacity and ferrous ions and ferrozine to measure iron-chelating ability. Results showed that HM, GIF and CIF all had significant Fe^3+^ reduction properties, while HMOs, GMOs and CMOs were all much less effective, relative to the control (Figure 2A). Also, GMOs had a slightly lower capacity to reduce Fe^3+^ compared with HMOs and GMOs (*p* < 0.0001). In terms of their iron-chelating abilities, HM showed a higher activity than that of GIF or CIF (*p* < 0.001) while GMOs and HMOs had a greater capacity to chelate Fe^2+^, relative to the control, compared to CMOs (Figure 2B). Interestingly, for the milk oligosaccharide fractions, HMOs and GMOs had significantly higher Fe^2+^ chelating abilities than those observed for CMOs. However, it is possible that other types of compounds in the UF filtrates, such as small-sized proteins like lactoferrin, might also exhibit iron-reducing or iron-chelating activities.

### 3.2. Effects of Milk Oligosaccharides on Reducing Inflammatory Cytokine Expression

**Figure 3:**
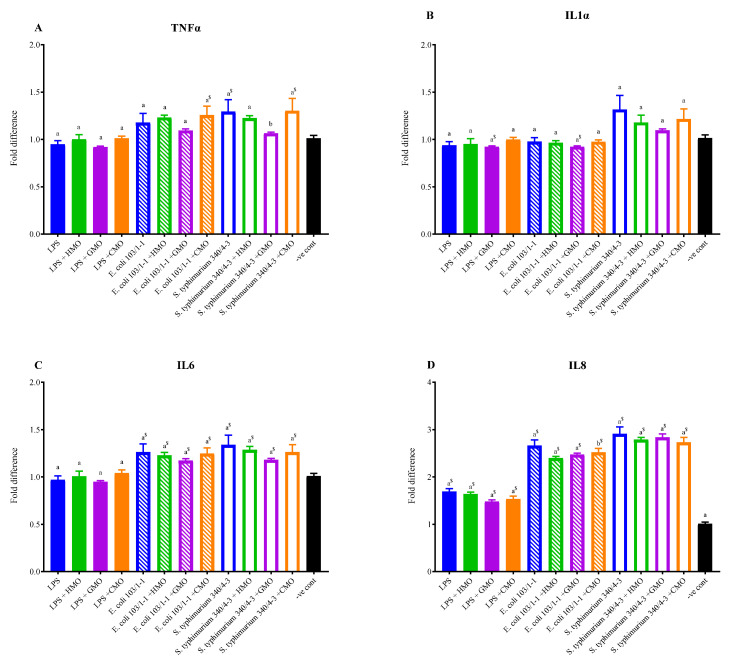
Gene expression of tumor necrosis factor-α (TNF-α) (**A**), interleukin 1-α (IL-1α) (**B**), interleukin 6 (IL-6) (**C**) and interleukin 8 (IL-8) (**D**), relative to that of glyceraldehyde-3-phosphate dehydrogenase (GAPDH), a housekeeping gene, following incubation of U937 cells with either water (negative control; black bars), LPS (filled colored bars), *E. coli* live cells (bars with diagonal lines) or *S. typhimurium* live cells (open bars) for 3 h, and then incubation for a further 3 h with no additions (blue bars), HMOs (green bars), GMOs (purple bars) or CMOs (orange bars). Bars with different superscripts (a or b) denote significant differences between treatments (*p* < 0.05). A $ superscript indicates values were different (*p* < 0.05) compared with the negative controls. See Figure 1 for sample abbreviations.

In in vitro experiments with uninduced U937 monocytic cells, stimulation with LPS failed to induce significant (*p* < 0.05) upregulation of TNF-α, IL-1α or IL-6, measured by determining their RNA transcript levels relative to the controls (Figure 3A–C). However, there was a significant upregulation of IL-8 (Figure 3D). Addition of *E. coli* bacteria (10^7^ live cells per assay), on the other hand, provided better stimulation (higher cytokine release), with significant (*p* < 0.05) upregulation of IL-6 and IL-8, but not TNF-α and IL-1α. The greatest effect was seen when live *S. typhimurium* bacteria were added; in this case three of the four cytokines were significantly upregulated, namely IL-6, IL-8 and TNF-α, with maximum expression for IL-8 approximately three-fold relative to the control (Figure 3D). Although some upregulation of IL-1α also occurred with *S. typhimurium*, it was not statistically significant, possibly because of the higher variation in the readings.

In cells where inflammation had been induced, treatment with HM or IF oligosaccharides for 3 h did not result in any statistically significant reductions in cytokine gene expression. Nonetheless, maximum reductions in expression of TNF-α, IL-1α and IL-6 were seen following addition of GMOs, relative to HMOs or CMOs. Repeat experiments with larger sample sizes and more highly purified oligosaccharide fractions are needed to confirm and refine these observations.

## 4. Discussion

The goal of this study was to understand the health properties of oligosaccharides in IF formulations, without addition of HMOs, compared to properties of HM and HMOs. Results demonstrated that HM, CIF and GIF had significant antioxidant capacities, with variable results observed for oligosaccharides (Figure 1 and Figure 2). Although the oligosaccharides had no significant effect (*p* > 0.05) on reducing the expression of proinflammatory cytokines, GMOs consistently reduced the expression levels of TNF-α, IL-1α and IL-6 to a greater extent than did CMOs or HMOs (Figure 3) and showed greatest in vitro antioxidant activity.

Other studies have shown that HM and other mammalian milks reduce oxidative stress and prevent DNA damage in vitro [23,37,38,39,40]. This is likely due to protein components in milk, such as casein and thioredoxin, peptides and reducing amino acids, and lactoferrin, that scavenge superoxide anions and reactive oxygen species [39,40,41,42,43]. In our study, GIF had the greatest antioxidant activities, surpassing those of HM and CIF. This agrees with the study of Lugonja et al. [44], who found that IFs had higher DPPH scavenging ability than that observed for HM. In addition to milk proteins and other components [23], the higher antioxidant activity of IFs is due to the presence of added antioxidants such as vitamins C and E [45]. The ability of 2′-FL, the most abundant HMO [46], to protect against intestinal inflammation by reducing oxidative stress has also been demonstrated in a mouse animal model. This effect appeared to be mediated by metabolites (mainly pantothenol) resulting from fermentation of this HMO by gut bacteria [47].

In this study, the highest radical scavenging ability occurred for GIF and GMOs, compared to activities of HM or CIF or their oligosaccharide fractions (Figure 1B,D). Goat’s milk more closely resembles HM than cow’s milk, including its GMOs [21], which vary according to breed [48,49,50]. The higher antioxidant activity of GMOs may be due to the unique suite of oligosaccharides found in goat’s milk. This observation, however, is at variance with the study of El-Fattah et al. [23], who found that goat and cow milks had roughly equivalent antioxidant capacities. The IFs used in our study were also fortified with vitamin supplements, and thus the possibility exists that some of these compounds may have contaminated the oligosaccharide filtrates and contributed to antioxidant effects observed for GMOs and CMOs. A relatively lower antioxidant effect of HMOs prepared as filtrates may also be explained by some HMOs being lost in retentates following 10 kDa molecular weight cut-off (MWCO) filtration [51].

While there have been several studies on the antioxidant properties of HM, CIFs and GIFs [14,40,52], to our knowledge, none has focused on cow or goat milk oligosaccharides. As noted by Vieira et al. [52], the structure and solubility of oligosaccharides affect their antioxidant activities. This may partly explain the observation that whilst HM contains the highest concentration, diversity and complexity of oligosaccharides, the free radical scavenging ability of HMOs as seen in our assays was less than that observed for GMOs (Figure 1A,D). In the study by Nijman et al. [16], the maximum oligosaccharide (galacto-oligosaccharides) concentration in five reconstituted commercial CIFs was 4.45 g/L, versus HMOs in HM at a level of 6–9 g/L. By comparison, van der Toorn et al. [50] indicate that GMOs in goat’s milk are about 100 times less in concentration (60−350 mg/L) than HMOs in HM, but still exceed that for CMOs in cow’s milk. The Oli6^®^ Stage 1 GIF used in this study contained 14 milk oligosaccharides at a concentration of 21 mg/L excluding 7 minor oligosaccharides [21]. Furthermore, our results showed that GMOs and HMOs have stronger iron-chelating ability than CMOs (Figure 2B). As noted in relation to antioxidant activity, this may be due to higher concentrations and/or differences in the structure of oligosaccharides present in goat’s milk.

Anti-inflammatory effects of oligosaccharides, especially HMOs, in vitro, and in animal models have been reported in several studies [33,53,54,55,56]. The objective of our study was to determine if oligosaccharides were able to attenuate inflammation after it had occurred using an in vitro model. Our results showed that HMOs, GMOs and CMOs had little effect in this regard (Figure 3), although GMOs consistently had relatively the greatest impact. Whether this was due to unique oligosaccharide structures present in GIF or for some other reason is not known. Apart from in vitro studies, there is limited evidence based on clinical trials to suggest that oligosaccharide-supplemented IFs can modulate the infant immune system, but this requires further investigation [4].

Oligosaccharides have been suggested to mediate anti-inflammatory effects through direct interactions with receptors on immune cells, or by acting as decoy receptors for pathogens, or by modulating the composition of the gut microflora and hence its metabolite complement [5,11]. The ability of certain oligosaccharides to bind to Toll-like receptors (TLRs) that are essential components of innate immunity has also been reported [57]. It is therefore likely that once LPS or pathogens have bound to immune cell receptors, oligosaccharides would not outcompete these for attachment to mediate any significant immune effect. This would explain the lack of response seen in the present study, since oligosaccharides were added after the agonists. Alternatively, it could be that certain necessary immune pathways in the U937 cells had not been activated.

Further studies are needed using better in vitro model systems, such as transwell plates, where intestinal epithelial cells and immune cells can be combined to simulate the gut environment [11,55] and where oligosaccharide fractions have been highly purified and characterized. Controlled dose-response studies with precisely measured concentrations of oligosaccharides would also provide more definitive answers about the inflammation attenuating-role of milk oligosaccharides.

In summary, this study showed that GIF (with GMOs being a likely major contributor) is superior compared to HM and CIF in terms of its antioxidant potential. Milk oligosaccharides from HM, GIF and CIF had an insignificant impact on iron chelation. HM, IFs and their oligosaccharides had limited ability to attenuate LPS- or pathogen-induced inflammation in vitro. Notwithstanding, GIF and GMOs appeared to be slightly better in this regard.

## 5. Conclusions

In vitro studies showed that goat’s milk infant formula (GIF) and its purified oligosaccharides were superior to cow’s milk infant formula (CIF) and human milk (HM), and their oligosaccharide fractions, in terms of their ability to neutralize oxidative radicals. HM, CIF and GIF showed limited in vitro inflammation-attenuating effects following induction of inflammation in cell culture. Further studies are needed to verify these observations. The results suggest that goat’s milk is a potentially useful source of oligosaccharides for new IF formulations, in addition to the permitted synthetic HMOs, for improved infant gut health.

## Data Availability

The raw data supporting the conclusions of this article will be made available by the authors on request.
